# *In Vitro *Antimicrobial and Antioxidant Activities of Ethanolic Extract of Lyophilized Mycelium of *Pleurotus ostreatus* PQMZ91109

**DOI:** 10.3390/molecules17043653

**Published:** 2012-03-26

**Authors:** Emanuel Vamanu

**Affiliations:** Faculty of Biotechnology, University of Agronomical Sciences and Veterinary Medicine Bucharest, 59 Marasti blvd, Bucharest 011464, Romania; Email: email@emanuelvamanu.ro

**Keywords:** mushroom, mycelium, antimicrobial, antioxidant, phenols, flavonoids

## Abstract

The antioxidant and antimicrobial potential of the ethanolic extract of *Pleurotus ostreatus* PQMZ91109 mycelium was determined based on inorganic and organic nitrogen sources in the culture medium. The presence of ammonium sulfate resulted in a greater accumulation of bioactive compounds compared with the organic ones. This finding was also confirmed by the low values of the ascertained EC_50_ and minimum inhibitory concentration (MICs). Among the organic sources, peptone followed by corn extract, led to a more important radical-scavenging activity. The extracts selectively inhibited the tested strains, mainly the two of the genus *Candida*, at an MIC value of 1.25 mg/mL. The antioxidant potential was evaluated by the inhibition capacity of the 2,2-diphenyl-1-picrylhydrazyl (DPPH) radical, β-carotene-linoleic acid, which is the reducing power. In addition, the quantity of the compounds with antioxidant effects confirmed the data obtained, they being present in the extracts.

## 1. Introduction

Edible mushrooms grow spontaneously on tree trunks or on decaying woody debris, in places with high humidity. Shiitake mushrooms such as *Lentinus edodes*, Maitake mushrooms such as *Grifola frondosa*, chanterelles such as *Cantharellus cibarius*, white button mushrooms such as *Agaricus bisporus*, and oyster mushrooms have shown that they serve as repositories of B-vitamins, organic acids, β-glucans, lipids, proteins, and micronutrients such as selenium or chromium [1]. Studies conducted to date reveal that the antimicrobial, antioxidant, and antitumor effects are due to the presence of these substances in aqueous or alcoholic extracts [2]. A special category of bioactive compounds is represented by the polysaccharides synthesized by these fungi. The obvious therapeutic effects of polysaccharides of the β-glucan type have emerged after undergoing cultivation in liquid medium [3]. Significant antioxidant effects of extracts from the mycelia of some mushrooms like *Coprinus comatus, Pleurotus ostreatus*, and *Leucopaxillus giganteus* were also highlighted by cultivation in liquid medium [4,5,6]. Consequently, it was recommended that the fungi and their derivatives can be used not only as dietary supplements, but also in medicinal products. Such products are used as an alternative to prevent and even treat diseases caused by the current lifestyle of mankind.

*Pleurotus* mushrooms, commonly known as oyster mushrooms, are quite easily cultivated artificially, most often in liquid medium. The oyster mushroom *P. ostreatus* is appreciated as a food due to its flavor and for its medicinal and bioremediational properties [7]. The species of the *Pleurotus* genus are considered to be an important source of dietary fiber and contain other important nutrients. Additionally, the antioxidant effect and the capacity to inhibit free radicals derive from the amount of phenolic compounds and flavonoids they contain [8,9]. Lyophilized extracts from *P. ostreatus* mycelium possess antioxidant and reducing activities which are higher than those of other commercial mushrooms. The antioxidant activities were positively correlated with the total polyphenol content, supported by lyophilization, a procedure that retains the highest quantities of these compounds [10]. Generally, freeze drying retains higher levels of phenolic content in samples than air drying [11,12]. The observed antimicrobial activity was classified as a defense mechanism against other organisms.

In the present study, the influence of an inorganic nitrogen source (ammonium sulfate) and of three organic sources (peptone, yeast extract, and corn extract) on the production of mycelium and on its functional properties was evaluated by determining the antioxidant activity, the reduction power, the inhibition of free radicals, and the antimicrobial activity. The antimicrobial activity was highlighted against some potentially pathogenic strains of Gram-positive and Gram-negative bacteria, as well as against two strains of the genus *Candida*. Also, determination of the amount of phenolic compounds, of flavonoids, and of the carotenoid compounds (β-carotene and lycopene) was carried out.

## 2. Results and Discussion

### 2.1. Effect of Nitrogen Source on Mycelia Growth

**Figure 1:**
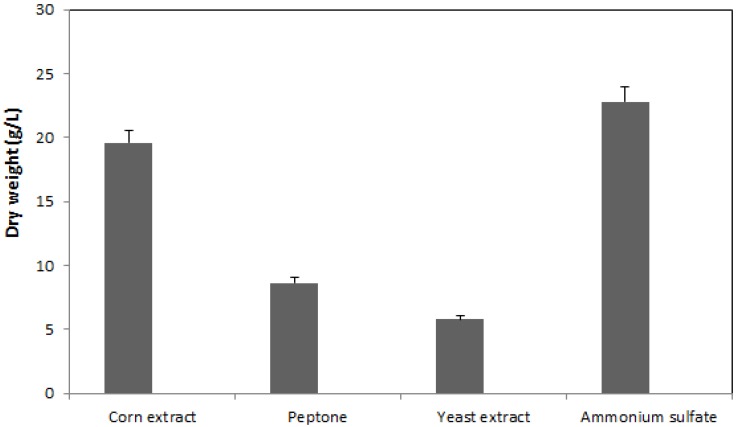
Effect of nitrogen source on mycelia growth of the *Pleurotus ostreatus* PQMZ91109. Values are expressed as mean ± SD (*n* = 3).

Use of an inorganic nitrogen source, ammonium sulfate, led to the best results, *i.e*., 22.77 g mycelium/L. For the three sources of organic nitrogen the order was: corn extract > peptone > yeast extract (Figure 1). In general, it can be appreciated that PQMZ91109 development was compact, with the density of the hyphae depending on the source of nitrogen used. It was observed that in the presence of corn extract the color of the hyphae was darker, more towards brown. In the other cases, the color was whitish—creamy. The difference between the presence of ammonium sulfate and corn extract in the culture medium was approximately 14%. The size of the colonies was small with an average diameter of less than 0.1 cm when ammonium sulfate and corn extract were used. Peptone and yeast extract are also known to be efficient nitrogen sources for fungal biomass production but essentially, the quantity obtained depends on the used strain [13]. A smaller colony diameter could be explain by the development of high mycelia density that, probably, consumes more time. A medium containing less nutrients prompts the colony to grow rapidly in search of nutrition [14].

Edible mushroom mycelium possesses a high antioxidant capacity. Current research is focused on determining the conditions for advantageous cultivation in order to obtain a mycelium possessing significant biological and pharmacological activities. The study performed on PQMZ91109 mycelium growing in submerged conditions is supported by other previous studies that were conducted under similar conditions, with special reference to the nitrogen source [15], to the stirring conditions, and to the length of cultivation [16]. These studies confirm that the inorganic nitrogen was the most suitable nitrogen source. Of the organic ones, the use of peptone is confirmed, but in addition, the study highlights the possible use of corn extract as a source of nitrogen that can be used to obtain mycelium with high antioxidant activity. As regards the peptone, the studies, likewise, confirmed the presence of strains such as *Coriolus versicolor* [17]. Ease of use and low cost price make it an ideal source of nitrogen for cultivation in bioreactors. Use of corn extract is also confirmed by its heterogeneous composition, which in addition to various growth factors, vitamins, contains a small amount of carbohydrates, of which sucrose registers the largest proportion [18].

### 2.2. Scavenging Effect on DPPH

**Figure 2:**
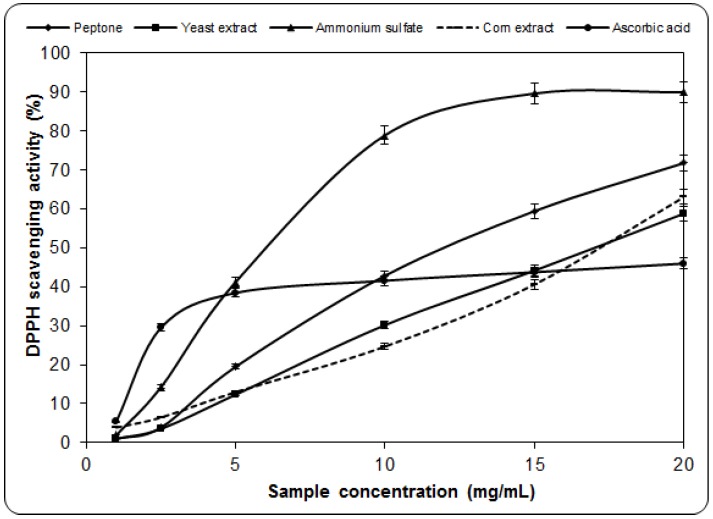
Scavenging activity of extracts from the *Pleurotus ostreatus* PQMZ91109 myceliumagainst 1,1-diphenyl-2-picrylhydrazyl. Values are expressed as mean ± SD (*n* = 3).

Stable DPPH radicals are widely used to evaluate the antioxidant activities of proton-donating substances according to their hydrogen-donating ability. DPPH radicals accept electrons or hydrogen radicals to form stable diamagnetic molecules. The antioxidant activity of substances can be expressed as the reduction capability of DPPH radicals at 517 nm [19,20]. The scavenging activity of radicals increased with increasing percentage of free radical inhibition [21]. The degrees of discoloration caused by the loss of the initial purple showed the potential for binding of the free radicals. The scavenging effect of the ethanolic freeze-dried PQMZ91109 mycelium on DPPH radicals increases with sample concentration, depending on the source of nitrogen used for the cultivation of the mycelium (Figure 2). Maximum values were obtained for mycelium grown in the presence of ammonium sulfate. The obtained order, depending on the source of nitrogen was: ammonium sulfate > peptone > corn extract > yeast extract. The scavenging activity of DPPH at a concentration of maximum 20 mg/mL ranged between 58.76 and 89.93%. Thus, the EC_50_ was 6.74 mg/mL for ammonium sulfate. In addition, ascorbic acid revealed a lower scavenging ability of 45.97%, at 20 mg/mL.

In the case of *Pleurotus squarrosulus*, similar percentages of DPPH scavenging activity, of 80–85% for aqueous extracts have been reported. In turn, the methanolic extract showed similar values. For PQMZ91109, these values are dependant on the nitrogen source. On the other hand, for *Agaricus blazei*, a scavenging ability of 97.4% is obtained, which means a difference of 7.6% [6] when compared with the maximum value obtained for PQMZ91109, at 20 mg/mL. For the ethanolic extracts of *G. frondosa* T1 and T2, the scavenged DPPH radicals were 99.19 and 84.36% at 20 mg/mL [22]. For *Leucoagaricus pudicus*, DPPH scavenging activity was 64.6%, and for *Amanita caesarea*, it was 79.4% in both cases for the same concentration of 20 mg/mL. Thus, it may be safely considered that for these species, both the alcoholic and aqueous extracts gave rise to the presence of significant amounts of antioxidant compounds. They react with DPPH radicals, the majority of them being reduced. The performance of ethanolic extracts of PQMZ91109 was higher than the standard ascorbic acid, which is in agreement with some previous studies [23,24].

### 2.3. Antioxidant Activity Against β-Carotene-Linoleic Acid

**Figure 3:**
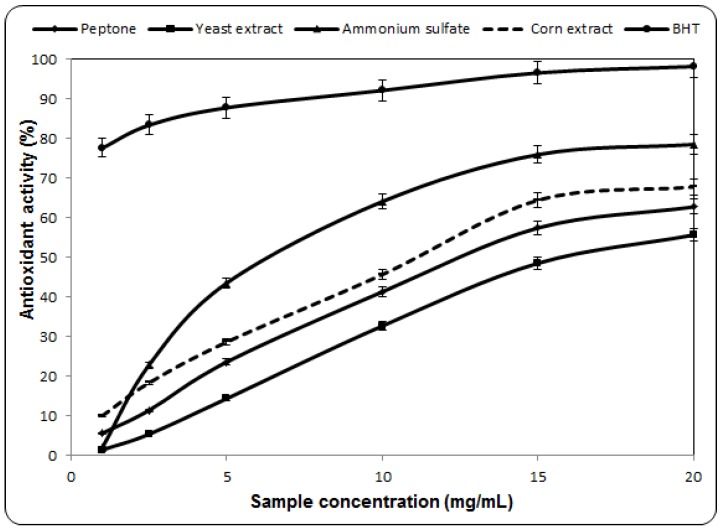
Antioxidant activity against β-carotene-linoleic acid of ethanolic extracts from *Pleurotus ostreatus* PQMZ91109. Values are expressed as mean ± SD (*n* = 3).

Polyunsaturated fatty acids, such as linoleic acid, are easily oxidized by oxygen in air. This auto-oxidation leads to the occurrence of chain reactions with the formation of coupled double bonds, and at a later stage also obtaining secondary products such as aldehydes, ketones, and alcohols [8,25]. Using this method to characterize the antioxidant capacity it was noticed that it increases with sample concentration. The most effective source of nitrogen was the inorganic one, ammonium sulfate. For the organic ones the order was: corn extract > peptone > yeast extract (Figure 3). If the EC_50_ is about 9.8 mg/mL for ammonium sulfate, for the corn extract it increases up to 13.8 mg/mL. In contrast, for BHT, the value is 0.2 mg/mL. The inhibition ratio corresponds to the previous studies of Barros *et al.* [4], which showed that the antioxidant activities of *L. giganteus* and *Agaricus arvensis* were 61.4 and 46.7% for concentrations below 10 mg/mL. Instead, the antioxidant activity of PQMZ91109 was 64.12% at 10 mg/mL for ammonium sulfate. The obtained values were outweighed by extracts from the fruiting bodies of *L. edodes* which had a value of more than 90% at a concentration of 8 mg/mL [26].

### 2.4. Determination of Reducing Power

In the reducing power assay, most antioxidant compounds convert the oxidized form of iron (Fe^3+^) in ferric chloride to ferrous (Fe^2+^) [27]. This is because the reducing capacity of a compound may serve as a significant indicator of its antioxidant potential, and the efficacy of certain antioxidants is known to be associated with their reducing power [5].

**Figure 4:**
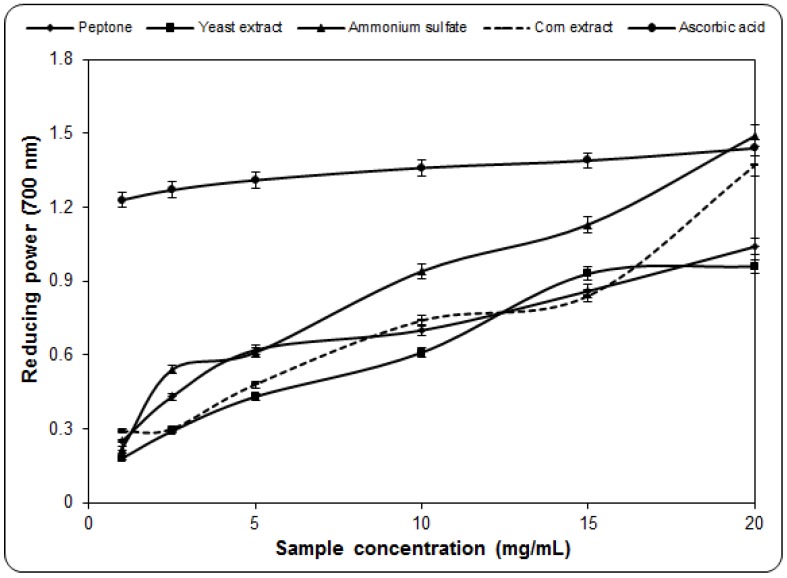
Reducing power of ethanolic extracts from the mycelium of *Pleurotus ostreatus* PQMZ91109. Values are expressed as mean ± SD (*n* = 3).

The results of this research showed that the reducing power of lyophilized mycelium obtained in the presence of ammonium sulfate yielded the best results (Figure 4), with a maximum value of 1.49 for a concentration of 20 mg/mL. From the resulting data obtained it was found that all extracts possess reduction capacity. EC_50_ values for the reducing power were 5.24, 7.7, 6.37, and 3.85 mg/mL for peptone, yeast extract, corn extract, and ammonium sulfate, respectively. In contrast, for ascorbic acid, the value was 0.085 mg/mL. Compared with *L. edodes* dry extracts, they were higher by about 60% for a 1 mg/mL concentration. Compared with the extracts of *Hericium erinaceum*, the difference was about 48% for the same concentration [28]. Thus, the obtained results indicated the presence of some significant amounts of reductone and ascorbic acid, which could react with free radicals to stabilize and block radical chain reactions. Compared with previous researches using the same species [5], valuable results have been obtained, which represent additional confirmation of the fact that the nitrogen source has a major influence not only on the amount of mycelium produced in liquid medium, but also on antioxidant properties of the alcoholic extracts.

### 2.5. Scavenging Effect on Hydroxyl Radicals

The hydroxyl radical is the most reactive of the reactive oxygen species, and it induces severe damage in adjacent biomolecules. The hydroxyl radical can cause oxidative damage to DNA, lipids, and proteins [5]. Thus, removing the hydroxyl radical is vital for the protection of living systems [29]. The ethanol extracts from PQMZ91109 mycelium display a scavenging activity according to concentration and nitrogen source, as shown in Table 1. Following the studies conducted, the hydroxyl radical-scavenging effect of the mushroom extract in a concentration of 20 mg/mL was 81.5% for the mycelium obtained in the presence of inorganic nitrogen source. In general, all extracts showed good hydroxyl scavenging activity. EC_50_ values for peptone and corn extract were very close with a difference of about 30% of that determined for ascorbic acid at 6 mg/mL used as the standard. Instead, for ammonium sulfate, the difference when compared with the standard was approximately 9%. The obtained data are consistent with those reported for the strain *P. squarrosulus* in the case of methanolic extract, but not for the aqueous ones where the EC_50_ value could drop by 51% [30]. For strains of the same species, the values of EC_50_ can reach 8 mg/mL [5] which is higher by about 30%, and which demonstrates the efficiency of mycelium produced by submerged culture.

**Table 1:** EC_50_ values for radical scavenging effect and bioactive compounds obtained from different mycelium extracts of *Pleurotus ostreatus* PQMZ91109. Values are mean ± SD of 3 separate determinations, each in triplicate.

	EC_50_ (mg/mL)			
	Peptone	Yeast extract	Ammonium sulfate	Corn extract
**Hydroxyl scavenging activity**	8.81 ± 0.06	14.47 ± 0.11	6.54 ± 0.04	8.54 ± 0.02
**Superoxide radical scavenging activity**	2.81 ± 0.18	4.71 ± 0.2	1.27 ± 0.86	1.65 ± 0.61
**Nitric oxide scavenging activity**	1.34 ± 0.4	3.78 ± 0.59	0.77 ± 0.47	1.22 ± 0.85
	**Bioactive Compounds**			
**Ascorbic acid (mg/100 g)**	19.34 ± 0.1	17.8 ± 0.05	20 ± 0.07	21 ± 0.02
**Total free phenolics (mg gallic acid/100 g)**	80 ± 0.12	66 ± 0.43	83 ± 0.1	71 ± 0.51
**Flavonoids (mg quercetin/100 g)**	450 ± 0.79	387 ± 0.41	531 ± 0.54	477 ± 0.3
**Lycopene (mg/100 g)**	-	0.048 ± 0.02	0.27 ± 0.02	0.32 ± 0.06
**β-carotene (mg/100 g)**	-	-	0.32 ± 0.12	0.44 ± 0.1
**α-tocopherol (mg/100 g)**	1.57 ± 0.58	1.8 ± 0.22	18.76 ± 1.07	28.59 ± 0.61

### 2.6. Scavenging Effect on Superoxide Radicals

Scavenging effect on superoxide radicals is important because it plays a significant role in the generation of other reactive oxygen species such as hydrogen peroxide, hydroxyl radical, or singlet oxygen in living systems [31,32]. The superoxide anions are free radicals common in living systems, being generated by auto-oxidation processes or by enzymes [33,34,35]. The results obtained, presented as EC_50_ index (Table 1), show that ethanol extracts from PQMZ91109 mycelium manifest inhibition on the generated superoxide (*p* < 0.05). It reaches up to 82.4% for ammonium sulfate for a sample of 20 mg/mL. For the corn extract and peptone, the EC_50_ value increased by about 30 and 55%, respectively. Again, yeast extract was the source of nitrogen that displayed the lowest scavenging effect. Comparing the obtained results with those of the standard for vitamin C resulted in PQMZ91109 mycelium extract exhibiting a strong scavenging activity. As standard, the EC_50_ was lower with approximately 34%. The obtained data correspond to previous results obtained with recognized medicinal herbs, for example, extracts from seeds of *Psoralea corylifolia*. The ethanol-water extract had a capacity of superoxide radical inhibition of 83.3% [34]. Compared with ethanolic extracts of the algae *Ulva fasciata* and *Gracilaria salicornia*, the results were similar, except those for which the sample concentration was at half strength [36].

### 2.7. Scavenging Activity of Nitric Oxide

A high concentration of nitric oxide is associated with many diseases. Oxygen can react with nitric oxide leading to the appearance of nitrite and proxy nitrite anions which are other categories of free radicals [37]. Ethanolic extracts showed a strong inhibition effect of nitric oxide, with a maximum of 96.31% for the extract of mycelium grown in the presence of inorganic nitrogen source (*p* < 0.05). For organic nitrogen sources, the obtained order, depending on the scavenging activity, was: corn extract > peptone > yeast extract. Thus, the concentration of the mycelium extract, which achieved a 50% inhibition, was approximately 0.77 mg/mL for the ammonium sulfate. Instead, according to the presented order, the increase in the sample concentration was approximately 37, 42.5, and 80%, respectively. Root extracts of *Hedranthera barteri* presented an EC_50_ value of 0.47 mg/mL, and the curcumin, used as standard, presented a value of 0.04 mg/mL [38]. Compared with Moroccan truffles, where the EC_50_ was 0.25 mg/mL, in the case of the extract from PQMZ91109 lyophilized mycelium it was at least 67.5% higher for ammonium sulfate and with a minimum 79.5% for organic nitrogen sources [39].

### 2.8. Effect of Nitrogen Source on Antimicrobial Activity

The antimicrobial susceptibility testing conducted against target microorganisms is presented in Table 2. In general, the mycelium obtained in the presence of the four nitrogen sources showed a strong antimicrobial activity. Differences are also due to the type of organism tested. This low value of MIC indicates that these components possess promising potential to inhibit some of the antibiotic-resistant bacteria [40].

**Table 2:** Antimicrobial activity of mycelia extracts obtained by using four different nitrogen sources. Values are mean ± SD of 3 separate determinations, each in triplicate.

Nitrogen source	MIC (mg/mL)						
	*Escherichia coli *CBAB 2	*Bacillus cereus *CMGB 215	*Listeria innocua *CMGB 218	*Candida sp*.ICCF15	*Candida albicans* ATCC 20231	*Pseudomonas aeruginosa ATCC* 15442	*Staphylococcus aureus* ATCC 6588
**Corn extract**	1.25	12.5	20	1.25	1.25	12.5	12.5
**Ammonium sulfate**	-	2.5	2.5	1.25	1.25	2.5	12.5
**Yeast extract**	-	2.5	20	1.25	1.25	20	12.5
**Peptone**	-	5	2.5	1.25	1.25	2.5	12.5

The results were recorded as presence or absence of inhibition zones. The inhibitory zones indicated the absence of bacterial growth reported as positive, and the absence of the inhibitory zone reported as negative [41,42]. The two yeast strains were strongly inhibited by all extract types, resulting in an MIC value of 1.25 mg/mL. *Escherichia coli* CBAB 2 proved to be resistant to these extracts, except that which was obtained from mycelium grown in the presence of corn extract. *Staphylococcus aureus* ATCC 6588 was found to require a concentration of 12.5 mg/mL extract, regardless of the source of nitrogen used, for the occurrence of high antimicrobial activity. In contrast, *Pseudomonas aeruginosa* ATCC 15442 and *Listeria innocua* CMGB 218 were strongly inhibited by the mycelium extract grown in the presence of peptone and ammonium sulfate. In general, the inorganic nitrogen source, ammonium sulfate, resulted in obtaining a mycelium of which ethanolic extract has shown the most pronounced antimicrobial effect. The obtained results are contrary to those presented by Barros *et al*. [4] regarding the mycelium of *L. giganteus*, the extract of which requires a concentration of at least 20 mg/mL to inhibit the growth of yeasts of the genus *Candida*. Instead, they are in agreement with the findings regarding *E. coli* strain which also possesses a high resistance requiring a minimum of 20–25 mg/mL in the mentioned study.

### 2.9. Antioxidant Components

Table 1 presents the quantities of the most important components of fungi extracts that had a major contribution to the antioxidant effect and antiradical activities. Ascorbic acid is reported to directly interact with radicals in plasma, thus preventing damage to red cell membranes [5]. In this case, the difference was determined by the nitrogen source used for mycelium growth.

Thus, the amount of ascorbic acid ranged from 17.8 to 21 mg/100 g of freeze-dried extract. The obtained amount was significant for this genus, in which a maximum of 25 mg/100 g was achieved [5]. Compared with species of the genus *Agaricus* and *L. edodes*, the results were similar, falling within the presented limits [43]. Compared with the studies of Barros *et al*. [4] regarding wild edible Portuguese mushrooms, PQMZ91109 content can be lower at 44% [10].

Phenolic compounds derived from plants are a favorite research target because of their possible use as dietary supplements or food preservatives [5]. In the case of mushrooms, they represent the main antioxidant component. For PQMZ91109, the obtained amount was very high, being double compared to that obtained for *L. giganteus* strain in the presence of KNO_3_ as an inorganic source of nitrogen [44]. Compared with ethanol extracts from *P. ostreatus* freshly harvested whole mushrooms, in the case of the mycelium obtained in liquid medium the total amount of phenols was at least 10 times higher [5]. The minimum quantity of 66 mg gallic acid/100 g was obtained for yeast extract. The maximum accumulated amount of 83 mg gallic acid/100 g, was obtained for ammonium sulfate. In this case, the total quantities of phenols obtained were higher even in comparison with *Ganoderma lucidum*, which was 55.96 mg/g [45].

The amount of flavonoids in the mycelium extracts obtained in the presence of four different nitrogen sources, confirmed that the inorganic source, ammonium sulfate, provided the best results. For the organic source, the order was: corn extract > peptone > yeast extract (*p* < 0.05). The results correspond to those previously carried out by Ameer and Al-Laith [39] with *Tirmania nivea*. The obtained values correspond to some previous studies with *L. edodes*, where an amount of 410 mg/g was reported [46]. Compared with extracts of some medicinal herbs like *Echinacea purpurea* L, with 86 mg quercetin/g, the obtained quantities were a consequence of the growing PQMZ91109 mycelium in liquid medium, and of the importance of the nitrogen source [47].

Carotenoids represent a major category of antioxidants, being known to bestow health benefits. Carotenoids are important because they have an active role in the protection process of human body cells, serving to balance and offset the destructive effects of free radicals. They are also natural colorants and stabilizers [5]. PQMZ91109 mycelium extracts had a low β-carotene and lycopene content. These two carotenoid compounds were absent in the case when using peptones. Of organic nitrogen sources, only the corn extract caused the obtaining of an amount of 44 mg/100 g of β-carotene. This was approximately 27% higher than that in the case of ammonium sulfate. The same situation was found for lycopene, but the difference was higher at 15.6%. Compared to studies of Jayakumar *et al*. [5], the results were very low, as they reported for *P. ostreatus* β-carotene amounts of 3.1 mg/100 g. Instead, the results were in agreement with those obtained for the strain *P*. *squarrosulus* in aqueous and/or alcoholic extracts [30].

α-Tocopherol is a classical and important antioxidant, well known as a scavenger of free radicals. It possesses anti- and pro-oxidant properties that act against low density lipoproteins, ensuring protection of the blood vessels and prevention of cardiovascular diseases [48]. In this study, α-tocopherol concentration ranged from 1.57–28.59 mg/100 g extract. The maximum amount was identified in the mycelium extract cultivated in the presence of corn extract. Thus, it is demonstrated once again the important role of the nitrogen source in the presence of compounds with antioxidant effect.

Owing to the increasing demand for natural bioactive compounds in the pharmaceutical and food industries, the interest in fungi has risen steadily in recent years. Since the fungus mycelium contains a significant amount of vitamins, fibers, phenolic compounds, and carotenoids, research interests focused on the determination of antioxidant capacity [18]. In mushroom extracts, antioxidant capacity is mainly determined by the amount of phenolic compounds they contain. Their quantity also influences the inhibition capacity of the free radicals, which, in the case of PQMZ91109, was correlated with the used nitrogen source and compared with the extracts of other mushrooms and medicinal herbs. In addition, it was confirmed that the lyophilization of the mycelium, and mainly of the final extract, determined the preservation of the maximum amount of phenols and flavonoids, which was in agreement with studies performed on some medicinal plant extracts [15,49].

The correlation between antioxidant compounds and freeze-dried PQMZ91109 mycelia was also evaluated. The *R*^2^ coefficient had good values between the content of total phenolics, flavonoids and ascorbic acid with antioxidant properties, depending on the nitrogen sources: for DPPH scavenging activity (*R*^2^= 0.783–0.9606), nitric oxide scavenging activities (*R*^2^= 0.845–0.991), superoxide radical scavenging activity (*R*^2^ = 0.788–0.9279), hydroxyl scavenging activity (*R*^2^ = 0.790–0.8979), reducing power (*R*^2^ = 0.888–0.9369) and antioxidant activity (*R*^2^= 0.768–0.948). These data are in accordance with other researches which demonstrated the major contribution of polyphenols to the antioxidant activity of freeze-dried extracts [50]. However, the amount of carotenoids could not exhibit a positive correlation with antioxidant properties. Moreover, antioxidant activities of extracts are correlated to α-tocopherol contents. The correlation coefficient varied from low to significant (*R*^2^ = 0.307–0.738).

The highest content of bioactive compounds of the mycelium extracts could explain the measured MIC values. A direct correlation between phenol content and antimicrobial properties was observed for all four nitrogen sources. Ammonium sulfate showed better results than organic nitrogen sources, expressed by lower values of MICs, which was in agreement with the higher content of bioactive compounds found. Because of its lower content in bioactive compounds, the mycelium cultivated in the presence of yeast extract was the less effective (higher MICs), not showing activity against *Escherichia coli *CBAB 2. The same behavior was observed for the other nitrogen sources, except corn extract. The correlations between antimicrobial activity and total phenolic content, expressed by *R*^2^ values, were between 0.34 and 0.897 [51,52].

## 3. Experimental

### 3.1. Chemicals

All chemicals and reagents were purchased from Sigma Aldrich GmbH (Sternheim, Germany). All other unlabelled chemicals and reagents were of analytical grade.

### 3.2. Culture and Storage Condition

The mushroom *P. ostreatus* PQMZ91109 was isolated from the stem of a poplar (Băneasa forest, Romania) and was authenticated by D. Pelinescu (Faculty of Biology, University of Bucharest, Bucharest, Romania). The mycelia were maintained on potato dextrose agar (PDA) at 4 °C. The microorganisms were subcultured at regular intervals (45 days) to maintain viability.

### 3.3. Media Preparation and Fermentation Condition

The fungi were initially grown on PDA medium for 10 days at 25 °C. The inoculum was prepared by growing mycelium on a LabTech rotary shaker at 150 rpm for 5 days and at 25 °C in 500 mL Erlenmeyer flasks containing 250 mL of the following synthetic medium (per liter): 6.0 g glucose, 100.0 g malt extract, 20.0 g yeast extract, 1.0 g KH_2_PO_4_, and 0.5g MgSO_4_ × 7H_2_O. The medium was adjusted to pH 5.5 with 0.2 M NaOH [53].

Submerged fermentation was carried out in 1,000 mL Erlenmeyer flasks, containing 700 mL of liquid medium (KH_2_PO_4_ 0.2%, CaSO_4_ 0.5%, MgSO_4_ 0.05%, and Na_2_HPO_4_ 0.01% in 5% extract solution of corn flour) and was performed with 4 different nitrogen sources: corn extract (dry substance 40%), peptone, yeast extract, and ammonium sulfate. The nitrogen sources were added as 10 g/L. The inoculated flasks were maintained on a LabTech rotary shaker at 150 rpm and 25 °C. After 7 days of growth, the mycelium was recovered from the liquid medium by centrifugation at 3,500× g for 10 min. Next, the obtained mycelia were washed 3 times with distilled water and freeze-dried in an Alpha 1-2 LD freeze-dryer in the absence of a cryoprotective agent [4,54].

### 3.4. Preparation of Mushroom Extract

The extract was obtained by ethanol extraction of freeze-dried mushroom mycelia. Ethanol extraction of the mycelia was accomplished by stirring at 150 rpm for 24 h at 20 °C, with a ratio of 1 g of freeze-dried biomass per 10 mL solvent. The broth was centrifuged at 3,500× g for 10 min and the supernatant was filtered using Whatman No. 1 filter paper. The ethanol extract was freeze-dried. The freeze-dried extract was then re-dissolved in 80% ethanol (v/v) to yield solutions containing 1.0, 2.5, 5.0, 10.0, 15.0, and 20.0 mg of extract per mL.

### 3.5. Antimicrobial Activity

*In vitro* antimicrobial susceptibility tests were performed using a panel of microorganisms from the collection of the Faculty of Biotechnology, Bucharest, Romania: Gram positive bacteria (*Listeria* *innocua *CMGB 218, *Bacillus cereus *CMGB 215, Staphylococcus aureus ATCC 6588), Gram negative bacteria (*Escherichia coli *CBAB 2, Pseudomonas aeruginosa ATCC 15442), and yeast (Candida albicans ATCC 20231, *Candida sp*. ICCF15). The yeast and bacteria were maintained in 20% glycerol and maintained at −80 °C.

#### Determination of Minimum Inhibitory Concentration (MIC)

The standard agar dilution protocol with doubling dilution was used. The extract was incorporated into nutrient agar at concentrations ranging from 0.39 mg/mL to 25 mg/mL. A control without the extract was also prepared. Ten μL of each test organisms, previously diluted to 10^6^ CFU/mL, were used to inoculate the plates. These were incubated at 37 °C for 24 h in the first instance, and for another 24 h before the growth was observed and recorded. The minimum inhibitory concentrations (MICs) of the extract for each test microorganism were considered the agar plate with the lowest concentrations without growth [55].

### 3.6. Determination of Antioxidant Activities

#### 3.6.1. 1,1-Diphenyl-2-picrylhydrazyl Radical Scavenging Activity of Freeze-Dried Mushroom Extracts

The reaction mixture contained 50 μL of test samples (or 80% EtOH as a blank) and 5 mL of a 0.04% (w/v) solution of DPPH in ethanol. Discoloration was measured at 517 nm after incubation for 30 min. DPPH radical concentration was calculated using the following equation: DPPH scavenging effect (%) = (A_0_ − A_P_)/A_0_ × 100, where A_0_ was the absorbance of the control and A_P_ was the absorbance in the sample. The actual decrease in absorption induced by the test compounds was compared with the positive controls. Ascorbic acid was used for comparison and as a positive control. The extract concentration providing 50% of free radical scavenging activity (EC_50_) was calculated from the graph of the radical scavenging activity (RSA) percentage against extract concentration [56,57].

#### 3.6.2. Antioxidant Activity by β-Carotene-Linoleic Acid

The antioxidant activity was determined with slight modifications of the procedure previously described by Sokmen *et al*. [58] Briefly, β-carotene (0.5 mg) was dissolved in chloroform (1 mL) and linoleic acid (25 μL) and Tween 40 (200 mg) were added. Chloroform was removed using a rotary vacuum evaporator and distilled water saturated with oxygen (100 mL) was added with vigorous shaking. A volume of 2.5 mL of this reaction mixture was dispensed into test tubes and 350 μL of various concentrations of the extracts were added. The absorbance was immediately measured at 490 nm. The reaction mixture was incubated at 50 °C for 2 h and the absorbance was measured again. The same procedure was repeated with a synthetic antioxidant (α-tocopherol [TOC] and butylated hydroxytoluene [BHT]) and a control. Inhibition ratio of linoleic acid oxidation was calculated for the test samples and for the synthetic antioxidants [59].

#### 3.6.3. Reducing Power of Freeze-Dried Extracts

Reducing power was determined according to the method described by Gulcin *et al*. [60] Each extract (in 2.5 mL of ethanol) was mixed with 200 mM sodium phosphate buffer (2.5 mL, pH 6.6) and 1% potassium ferricyanide (2.5 mL), and the mixture was incubated at 50 °C for 20 min. Next, 10% trichloroacetic acid (2.5 mL) was added, and the mixture was centrifuged at 3,000 g for 10 min. The upper layer (2.5 mL) was mixed with deionized water (2.5 mL) and 0.1% ferric chloride (0.5 mL). Finally, the absorbance was measured at 700 nm and compared to a blank. The extract concentration providing 0.5 of absorbance (EC_50_) was calculated from the graph of absorbance at 700 nm plotted against the extract concentration. Ascorbic acid was used as positive control [9,57].

#### 3.6.4. Superoxide Radical Scavenging Activity of Freeze-Dried Extracts

The scavenging activity on superoxide radicals was evaluated according to the methods described by Chou *et al*. [61]. The reaction mixture contained the same volume of 120 μM PMS (phenazine methosulfate), 936 μM NADH, freeze-dried extract, and 300 μM NBT, in a total volume of 1 mL of 100 mM phosphate buffer (pH 7.4). After 5 min of incubation at ambient temperature, absorbance of the resulting solution was measured at 560 nm. The superoxide radical activity was calculated as: scavenging effect (%) = (1 − absorbance of sample/absorbance of control) × 100. Ascorbic acid was used for comparison. EC_50_ value (milligram extract/mL) is the effective concentration at which hydroxyl radicals were scavenged by 50% [62].

#### 3.6.5. Hydroxyl Radical Scavenging of Freeze-Dried Extracts

Hydroxyl radical scavenging was assayed as described by Varshneya *et al.* [63] with a slight modification. The assay is based on the quantification of the degradation product of 2-deoxyribose by condensation with thiobarbituric acid (TBA). Hydroxyl radicals were generated using the Fe^3+^-ascorbate-EDTA-H_2_O_2_ system (the Fenton reaction). The reaction mixture contained, in a final volume of 1 mL: 2-deoxy-2-ribose (2.8 mM), KH_2_PO_4_-KOH buffer (20 mM, pH 7.4), FeCl_3_ (100 μM), EDTA (100 μM), H_2_O_2_ (1.0 mM), ascorbic acid (100 μM), and various concentrations (2–20 mg/mL) of the freeze-dried extracts or reference compound. After incubation for 1 h at 37 °C, the reaction mixture (0.5 mL) was added to 2.8% TCA (1 mL), then 1% aqueous TBA (1 mL) was added and the mixture was incubated at 90 °C for 15 min to develop the color. After cooling, the absorbance was measured at 532 nm against an appropriate blank solution. All tests were performed three times. Percentage inhibition was evaluated by comparing the test and blank solutions. EC_50_ value (milligram extract/mL) is the effective concentration at which hydroxyl radicals were scavenged by 50% [64].

#### 3.6.6. Nitric Oxide Scavenging of Freeze-Dried Extracts

Nitric oxide scavenging activity was measured spectrophotometrically. Sodium nitroprusside (5 mmol/L) in phosphate buffered saline, pH 7.4, was mixed with different concentrations of the extract prepared in ethanol and incubated at 25 °C for 30 min. A control without the test compound, but with an equivalent amount of ethanol, was also used. After 30 min, 1.5 mL of the incubated solution was removed and diluted with 1.5 mL of Griess reagent (1% sulphanilamide, 2% phosphoric acid and 0.1% *N*-1-naphthylethylenediamine dihydrochloride). Absorbance formed during diazotization of the nitrite with sulphanilamide and subsequent coupling with *N*-1-naphthylethylenediamine dihydrochloride was measured at 546 nm and the percentage scavenging activity was measured with reference to the standard (curcumin). The EC_50_ value (milligram extract/mL) is the effective concentration at which hydroxyl radicals were scavenged by 50% [65].

### 3.7. Determination of Antioxidant Component

#### 3.7.1. Determination of Total Phenolic Content

The content of total phenols was determined by spectrophotometry, using gallic acid as standard, according to the method described by the International Organization for Standardization (ISO) 14502-1. Briefly, an aliquot of the diluted sample extract (1.0 mL) was transferred in duplicate to separate tubes containing a 1/10 dilution of Folin-Ciocalteu’s reagent in water (5.0 mL). Then, a sodium carbonate solution (4.0 mL, 7.5% w/v) were added. The tubes were then allowed to stand at room temperature for 60 min before absorbance at 765 nm was measured against water. The content of total phenols was expressed as gallic acid equivalents in g/100 g extract. The concentration of polyphenols in samples was derived from a standard curve of gallic acid ranging from 10 to 50 μg/mL (Pearson’s correlation coefficient: *r^2^* = 0.9996) [66].

#### 3.7.2. Determination of Total Flavonoids

Sample (0.25 mL of different concentrations of the extracts) was added to a tube containing distilled water (1 mL). Next, 5% NaNO_2_ (0.075 mL), 10% AlCl_3_ (0.075 mL) and 1 M NaOH (0.5 mL) were added sequentially at 0, 5 and 6 min. Finally, the volume of the reacting solution was adjusted to 2.5 mL with double-distilled water. The absorbance of the solution at a wavelength of 410 nm was detected using the Helios λ spectrophotometers. Quercetin is a ubiquitous flavonoid, present in many natural extracts, used as standard to quantify the total flavonoid content. Results were expressed in microgram quercetin equivalents/100 g extract [67,68].

#### 3.7.3. Determination of β-Carotene and Lycopene

For β-carotene and lycopene determination, the dried ethanolic extract (100 mg) was vigorously shaken with an acetone-hexane mixture (4:6, 10 mL) for 1 min and filtered through Whatman No. 1 filter paper. The absorbance of the filtrate was measured at 453, 505, and 663 nm. β-Carotene and lycopene content were calculated according to the following equations: 

lycopene (mg/100 mL) = −0.0458 × A_663_ + 0.372 × A_505_ − 0.0806 × A_453_

β-carotene (mg/100 mL) = 0.216 × A_663_ − 0.304 × A_505_ + 0.452 × A_453_

The results are expressed as mg of carotenoid/g of extract [15].

#### 3.7.4. Determination of α-Tocopherol

The content was determined spectrophotometrically according to the method of Kivcak and Akay. The α-tocopherol content in the extracts was calculated from the regression equation of the standard curve [69].

### 3.8. Statistical Analysis

All parameters for antimicrobial and antioxidant activity were assessed in triplicate, and the results were expressed as mean ± SD values of 3 observations. The mean values and standard deviation were calculated with the EXCEL program from Microsoft Office 2010 package.

## 4. Conclusions

The extract of an oyster mushroom mycelium (*Pleurotus ostreatus*) can be an effective natural antioxidant product for food and pharmaceutical industrial applications. The mushroom mycelium contains many different bioactive compounds with diverse biological activity depending on how it is obtained. *Pleurotus* mycelium extracts have a strong inhibiting and other beneficial or therapeutic health effects. The ethanol extract of the PQMZ91109 lyophilized mycelium and various antioxidants used proved to exhibit antioxidant activity, reducing power, and free radical inhibition properties, being dependent on the sample concentration. The same feature was also observed for antimicrobial activity, the extracts being effective against some strains of pathogenic potential for humans. The study showed that the PQMZ91109 mycelium represents a major source of phenols, flavonoids and β-carotene, acting in addition for the prevention of some diseases of modern society. The obtained results showed that the properties of nutraceuticals depend on the nitrogen source and, therefore, the PQMZ91109 mycelium extracts can be used as a rich source of antioxidants in pharmaceutical-type products.
